# Predictors of Cardiovascular Autonomic Neuropathy in Patients with Type 1 Diabetes

**DOI:** 10.3389/fendo.2014.00191

**Published:** 2014-11-25

**Authors:** Lucianne Righeti Monteiro Tannus, Karla Rezende Guerra Drummond, Eliete Leão da Silva Clemente, Maria de Fátima Bevilacqua da Matta, Marilia Brito Gomes, on behalf of the Brazilian Type 1 Diabetes Study Group (BrazDiab1SG)

**Affiliations:** ^1^Unit of Diabetes, Department of Medicine, State University Hospital of Rio de Janeiro (UERJ), Rio de Janeiro, Brazil

**Keywords:** type 1 diabetes, cardiovascular risk, cardiovascular autonomic neuropathy, heart rate variability, chronic complications

## Abstract

Cardiovascular disease (CVD) is the leading cause of mortality in patients with type 1 diabetes (T1D). The cardiovascular autonomic neuropathy (CAN), although considered as an independent risk factor for CVD, remains underdiagnosed. The aim of this paper was to determine the prevalence, predictors of CAN in patients with T1D and its association with other chronic complications of diabetes. Patients with T1D underwent a clinical-epidemiological survey, had blood and urinary samples collected, performed ophthalmoscopic and clinical neurological examination and cardiovascular reflex tests. One hundred and fifty one patients with T1D, 53.6% female, 45.7% Caucasian, mean age of 33.4 ± 13 years, diabetes duration of 16.3 ± 9.5 years, and glycated hemoglobin levels of 9.1 ± 2% were evaluated. The prevalence of CAN in the studied population was 30.5%. CAN was associated with age (*p* = 0.01), diabetes duration (*p* = 0.036), hypertension (*p* = 0.001), resting heart rate (HR) (*p* = 0.000), HbA1c (*p* = 0.048), urea (*p* = 0.000), creatinine (*p* = 0.008), glomerular filtration rate (*p* = 0.000), urinary albumin concentration (*p* = 0.000), LDL (*p* = 0.048), free T4 (*p* = 0.023), hemoglobin (*p* = 0.01) and presence of retinopathy (*p* = 0.000), nephropathy (*p* = 0.000) and diabetic neuropathy (*p* = 0.000), the following symptoms syncope (*p* = 0.000), post prandial nausea (*p* = 0.042), early satiety (*p* = 0.031), sexual dysfunction (*p* = 0.049), and gustatory sweating (*p* = 0.018). In logistic regression model, it was observed that only resting HR, diabetic neuropathy, and retinopathy were independent associated with CAN. In conclusion, CAN is a common chronic complication of T1D affecting about 30% of the studied population and is associated with the presence of other chronic complications. Indicators of CAN included age, diabetes duration, hypertension, resting HR, diabetic neuropathy and retinopathy, and symptoms suggestive of autonomic neuropathy. This study confirms the importance of systematic and early screening for CAN.

## Introduction

Type 1 diabetes (T1D) is the most common endocrine disorder of childhood and adolescence affecting about 10–20 million people worldwide (1). Poor glycemic control is associated with the development of micro and macrovascular chronic complications leading to a major impact on morbidity and mortality (2). Cardiovascular disease (CVD) is the leading cause of mortality in patients with T1D (3–5).

Cardiovascular autonomic neuropathy (CAN) although considered a common diabetic chronic complication (6) and associated with increased cardiovascular morbidity and mortality, remains underdiagnosed (6, 7).

Depending on the methodology and diagnostic criteria, the prevalence of CAN ranges from 2.6 to 90% among patients with diabetes and its prevalence increases with age, diabetes duration, and inappropriate glycemic control (8, 9) and may be associated with peripheral neuropathy (up to 62.5% of cases) and the presence of other cardiovascular risk factors, such as hypertension, dyslipidemia, diabetic nephropathy and retinopathy, arterial stiffness, left ventricular hypertrophy, and diastolic dysfunction (9, 10).

Cardiovascular autonomic neuropathy has been identified as a strong predictor of CVD in both patients with T1D and type 2 diabetes (T2D). Patients with diabetes and CAN have 5-year mortality rates ranging from 16 to 53%, depending on its severity (8).

In the DIAD study (*Detection of silent myocardial ischemia in asymptomatic diabetic subjects*) conducted in patients with T2D, the presence of CAN, determined by abnormal Valsalva ratio, was a strong predictor of silent myocardial ischemia, regardless of the presence of traditional cardiovascular risk factors such as hypertension, age, sex, and smoking (11–13).

Data from the Action to Control Cardiovascular Risk in Diabetes (ACCORD) study showed that patients with CAN presented mortality rate approximately 1.55–2.14 times higher than patients without CAN (14).

Unfortunately, clinical symptoms of CAN appears late in the course of the disease, which makes the use of cardiovascular reflex tests critical for CAN diagnosis (15–17). However, the presence of symptoms suggestive of dysautonomia as syncope, postprandial hypotension, exercise intolerance, resting tachycardia, gastroparesis, diarrhea, urinary incontinence, and erectile dysfunction may suggest the presence of CAN and these patients should be investigated (7).

According to Toronto Consensus, the analysis of heart rate variability (HRV) using the variation of RR intervals of the electrocardiogram are sensitive and specific methods used for the early detection of CAN and seven parameters can be used to assess the presence of CAN: the three spectral analysis frequency bands [very low frequency (VLF), low frequency (LF) and high frequency (HF)] and four tests proposed by Ewing [heart rate (HR) response to the Valsalva maneuver, to deep breathing, and to standing and the blood pressure response to standing] (17, 18).

The consensus of Diabetic Neuropathy Conference held in San Antonio in 1992 (15), of the Toronto Diabetic Neuropathy (17, 18), and the Study Group on the Diabetic Neuropathy Italian Society of Diabetology (16), advocates the use of cardiovascular reflex tests (deep breathing test, orthostatic test, OH test, and Valsalva test) as the gold standard tests for the diagnosis of CAN.

The time and frequency-domain HRV parameters seem to be more sensitive and changes in HRV may occur earlier than alterations on cardiovascular reflex tests. Tannus et al. (19) showed that short-term spectral analysis of HRV (VLF, HF, and LF) despite being a low cost, non-invasive, easy to perform, and does not require active cooperation of patients, demonstrated poor reproducibility (20–24) being thus considered as additional tests for the diagnosis of NAC (15–17).

Considering the paucity of data in the literature on the prevalence of CAN in the Brazilian population with T1D, this study aimed to determine the prevalence of CAN in Brazilian patients with T1D and to identify clinical and laboratory factors associated with the presence of CAN.

## Materials and Methods

This study is a cross-sectional study conducted in patients with T1D attended in the diabetes unit of State University of Rio de Janeiro. Written informed consent for the study was obtained from all of the patients aged 18 years or older or from the parents or guardians of the patients younger than 18 years. The study was approved by each local center’s ethics committee.

The inclusion criteria were patients with T1D, defined by a physician based on the criteria of American Diabetes Association (ADA) and Brazilian Diabetes Society (BDS), which include age less than 40 years old at diagnosis, typical clinical presentation (weight loss, polyuria, polydipsia), or history of diabetic ketoacidosis or ketonemia and need to use insulin without interruption since the diagnosis; age ≥13 years and diabetes duration ≥5 years. Exclusion criteria were pregnancy, lactation, chronic alcoholism, congestive heart failure, cardiac arrhythmias, acute respiratory failure or severe obstructive lung disease, infections, and acute complications of diabetes.

The following clinical and demographic data were obtained through an interview during a clinical visit: sex, age (years), weight (kg), height (m), body mass index [BMI (Kg/m^2^)], systolic (sBP) and diastolic blood pressure [dBP (mmHg)], HR, ethnicity, age at diagnosis (years), duration of diabetes (years), smoking status, alcohol consumption, daily insulin dose, use of other medications, and associated diseases.

Body mass index was calculated by dividing weight (kg) by height squared (m^2^). Individuals aged ≥18 years and BMI ≥25 and 30 kg/m^2^, were considered as overweight and obesity respectively and in subjects <18 years overweight was defined if BMI ≥85th percentile and obesity if BMI ≥95th percentile, according with growth charts from Center for Disease Control CDC), respectively (25).

Hypertension was defined as sBP ≥140 mmHg and/or dBP ≥90 mmHg or on therapy for hypertension (26) and in subjects <18 years as sBP and/or dBP >95 percentile according to sex, age, and height (27).

The following laboratory variables were performed: fasting plasma glucose (FPG), postprandial glucose (PPG), glycated hemoglonin (HbA1c), total cholesterol, high density lipoprotein (HDL), triglycerides, uric acid, plasma creatinine, plasma urea, sodium, potassium, liver enzymes [aspartate (AST) and alanine aminotransferase (ALT)], creatine phosphokinase (CPK), thyroid stimulating hormone (TSH), free T4, cyanoconalamin, and C-reactive protein (CRP). The low-density lipoprotein (LDL) was calculated using the Friedewald formula (LDL = total cholesterol − HDL + Triglycerides/5), when the values of triglycerides did not exceed 400 mg/dl (28).

Screening for retinopathy by fundoscopy and nephropathy by albuminuria and determination of glomerular filtration rate (GFR) was performed.

The diagnosis of micro and macroalbuminuria was established by urinary albumin concentration of respectively 17–173 mg/dl and ≥174 mg/dl, at least two random urine specimen (29). The GFR was calculated from the chronic kidney disease epidemiology collaboration (CKD-EPI) (30) equation in patients aged >16 years and the Schwartz equation for children and adolescents and was considered normal if GFR >60 ml/min/1.73 m^2^ (31, 32).

Screening for sensory motor neuropathy was performed by the neuropathic disability score (NDS) and neuropathic impairment score (NIS) (33, 34).

The evaluation of CAN was performed according to the protocol described elsewhere (19). Briefly, the subjects were instructed to refrain from drinking alcohol- or caffeine-containing beverages and to engage in smoking cessation for a minimum of 8 h prior to testing and to refrain from strenuous exercise for at least 24 h preceding the examination. The exclusion criteria were fever in the last two days, emotional distress on the day before the autonomic tests and arrhythmias.

Autonomic tests were performed in the morning. Subjects were instructed to assume the supine position in a metabolic unit with a controlled temperature (23°C) after an overnight fast or at least 2 h after a light meal (containing a total of 300 Kcal) standardized by a dietitian.

After 5 min of rest in the supine position with the head elevated 30° and after 5 min of breathing spontaneously, an electrocardiogram (EKG) was recorded for 300 s using a computer. The EKG was analyzed by a mathematical algorithm (fast Fourier transform) and expressed in a diagram of oscillation amplitude (HR variations per second) versus frequency (Hz).

The autonomic tests are described below. After each test, a resting period of 1 min was scheduled to prevent influences by the previous tests.

### Deep breathing test

The subject was kept in the supine position for at least 1 min and then invited to start the test with a deep inspiration to the maximum total lung capacity for 5 s, which was followed by a forced expiration down to the residual volume for 5 s. The time to alternate the respiratory cycle is signaled directly to the patient by the operator. Such a respiratory cycle was repeated three times. The expiration:inspiration ratio (E:I ratio) was determined by the ratio between the longest and shortest RR intervals obtained during the expiration and inspiration cycles, respectively, and the highest E:I ratio was considered (16).

### Valsalva maneuver

Without taking a deep breath beforehand, the subject, who was in the supine position, was invited to blow with an open glottis into a mouthpiece connected to a manometer and to maintain a constant expiratory effort equivalent to an intraoral pressure of 40 mmHg for 15 s. This test induces a physiological tachycardia, which persists for a maximum of approximately 14 s. After this period, the expiratory straining is suddenly released, and the subject should breathe regularly and remain silent and motionless until the end of the test. An EKG was continuously recorded for 70 s, when the physiological bradycardia is observed. The Valsalva ratio was determined by the ratio between the longest and shortest RR intervals. The occurrence of facial flushing, plethora, and neck vein engorgement testify to the correctness of the maneuver (7, 24). The test was performed twice, and the highest Valsalva ratio was considered.

### Orthostatic test

After lying in the supine position for at least 5 min, the subject was invited to stand up quickly but remain relaxed with their arms at rest alongside the body and without speaking or moving until the end of the test (180 s after standing up). The 30:15 or maximum:minimum (max:min) ratio was determined by the ratio between the longest (maximum bradycardia at approximately the 30th beat) and shortest (maximum tachycardia at approximately the 15th beat) RR intervals.

### Orthostatic hypotension test

After at least 5 min of supine rest, the blood pressure was measured at baseline and after 3 min of standing. A drop in the systolic blood pressure that was higher than or equal to 20 mmHg was considered abnormal (7, 24).

The diagnosis of incipient and defined CAN was considered in the presence of one and two abnormal cardiovascular reflex tests, respectively (17, 35).

#### Statistical analysis

The statistical analysis was performed using Statistical Package for Social Sciences (SPSS) for Windows (version 13.0). The data are presented as mean ± SD and median (minimum–maximum). Comparison between numeric variables was performed using *t*-test and Mann–Whitney test for variables with normal or skewed distribution, respectively. The chi-square test was used to compare the frequencies of two categorical variables. A Pearson’s correlation coefficient was calculates when applicable. Adjustments were made for potential confounders. A binary logistic regression was performed with diagnosis of CAN (yes/no) as the dependent variable. The following independent variables were included: sex, age, diabetes duration, pulse pressure, resting HR, BMI, hypertension, presence of micro or macroalbuminuria, presence of diabetic neuropathy and retinopathy, HbA1c, urea, creatinine, LDL cholesterol, and hemoglobin. Odds ratios with 95% confidence intervals (CI) were performed when indicated. A two-sided *p*-value less than 0.05 was considered significant.

## Results

One hundred and fifty-one patients with T1D were evaluated. Clinical and demographic data for the study population are presented in Table 1.

**Table 1:** **Clinical and demographic data of the studied population**.

Variable	
*N*	151
Age (years)	33.4 ± 13
Gender, female (%)	81 (53.6)
Ethnicity, Caucasian (%)	69 (45.7)
Age at diagnosis (years)	17.2 ± 9.8
Diabetes duration (years)	16.3 ± 9.5
BMI (Kg/m^2^)	23.4 (13.7–37.9)
Abdominal circumference (cm)	79.8 ± 9.8
Hypertension, *n* (%)	36 (23.8)
Current smoker, *n* (%)	11 (7.3)
sBP (mmHg)	124.6 ± 16.6
dBP (mmHg)	75.1 ± 10.4
Heart rate (bpm)	79 (50–134)
HbA1c (%)	9.1 ± 2
Fasting glycemia (mg/dl)	170 (21–561)
Postprandial glycemia	223 ± 95
Total cholesterol (mg/dl)	176 (98–330)
Triglycerides (mg/dl)	79 (32–748)
HDL-c (mg/dl)	62.9 (32.7–165.8)
LDL-c (mg/dl)	94 ± 27.4
Lipid-lowering mediations, *n* (%)	49 (32.5)
BP-lowering medications, *n* (%)	55 (33.8)
Diabetic retinopathy, *n* (%)	55 (37.7)
Normoalbuminuria/microalbuminuria) macroalbuminuria, *n* (%)	110 (75.9)/32 (23.4)/1 (0.7)
Diabetic neuropathy, *n* (%)	44 (29.1)

*The data are presented as counts (percentage), means ± SD, or medians (minimum/maximum)*.

*BMI, body mass index; sBP, systolic blood pressure; dBP, diastolic blood pressure; BP, blood pressure; GFR, glomerular filtration rate*.

Table 2 presents the variables used for assessment of CAN in the study population and in subgroups of patients with and without CAN. The prevalence of CAN as assessed only by HRV (spectral analysis) was higher than the prevalence of CAN assessed by cardiovascular reflex tests and showed moderate agreement (kappa = 0.44/*p* = 0.074). Data about the prevalence of CAN according to the diagnostic criteria are shown in Table 3.

**Table 2:** **Variables used for assessment of CAN**.

Frequency domain variables	All patients	Presence of CAN	Absence of CAN	*p*
VLF (ms^2^)	395 (11.4–8159)	154.5 (11.4–1308)	676 (48.9–8159)	0.000
LF (ms^2^)	301 (2.1–4568)	50.5 (2.1–2899)	565 (37.6–4568)	0.000
HF (ms^2^)	244 (3.9–8381)	43 (3.9–6590)	463 (16.3–8381)	0.000
VLF (%)	38.4 ± 18.9	51 (12.1–89.5)	33.6 (7.9–84.5)	0.000
LF (%)	27.4 ± 11.9	24 ± 11.7	28.9 ± 11.8	0.019
HF (%)	29 (4.1–74.2)	19.8 (4.1–63.9)	30.2 (3.7–76.5)	0.003
LF/HF ratio	0.97 (0.1–27.9)	1.1 (0.1–8.4)	1 (0.1–27.9)	0.509
**TIME DOMAIN VARIABLES**				
RR min (ms)	676 (30–1127)	663.5 (502–1127)	688 (30–1052)	0.297
RR max (ms)	903 (597–12266)	760.5 (597–2854)	943 (734–12266)	0.000
RRNN (ms)	799 547–1378)	707 (547–1195)	817 (618–1378)	0.000
SDNN (ms)	32 (4–155)	14 (4–155)	40 (13–147)	0.000
RMSSD (ms)	23 (1–157)	8.5 (1–157)	30 (7–140)	0.000
CV (%)	4 (0.7–21.9)	2 (0.7–21.9)	5.1 (1.6–15.5)	0.000
TP (ms)	1185 (26–15880)	377.5 (26–10797)	2125 (192–15880)	0.000
pNN50	1.59 (0–77.1)	0 (0–13.2)	7.7 (0–77.1)	0.000
**CARDIOVASCULAR REFLEX TESTS (GOLD STANDARD)**				
Max–min ratio	1.36 (1.01–2.2)	1.15 (1.01–1.9)	1.44 (1.09–2.2)	0.000
Valsalva ratio	1.71 (1.04–3.74)	1.37 (1.04–2.54)	1.9 (1.39–3.74)	0.000
E:I ratio	1.36 (0.98–2.28)	1.1 (0.98–1.75)	1.45 (1.12–2.28)	0.000
Decrease in sBP (mmHg)	3 (−27–35)	7.5 (−27–35)	2 (−20–17)	0.000
Decrease in sBP ≥20 mmHg, yes, *n* (%)	10 (6.6)	10 (6.6)	0	0.000

*RR min, RR minimum; RR max, RR maximum; RRNN, mean RR intervals; SDNN, standard deviation of all normal to normal RR intervals; CV, coefficient of variation; RMSDD, the root mean squared successive difference between adjacent RR intervals; TP, total power; pNN50, percentage of intervals between consecutive normal beats that exceed 50 ms; VLF, very low frequency; LF, low frequency; HF, high frequency; E:I ratio, expiration to inspiration ratio; Valsalva ratio, Max: min ratio, maximum to minimum ratio; sBP, systolic blood pressure*.

*The data are presented as counts (percentage), means ± SD, or medians (minimum/maximum)*.

**Table 3:** **Prevalence of CAN according to the diagnostic criteria**.

	CAN prevalence		
	Total	NAC defined	NAC incipient
Cardiovascular reflex tests + HRV (spectral analysis), *n* (%)	53 (35.1)	39 (25.8)	14 (9.3)
Cardiovascular reflex tests, *n* (%)	46 (30.5)	17 (11.3)	29 (19.2)
HRV (spectral analysis), *n* (%)	61 (40.4)	51 (33.8)	10 (6.6)

*HRV, heart rate variability; CAN, cardiovascular autonomic neuropathy*.

The prevalence of autonomic symptoms in the general population, in patients with and without CAN is shown in Table 4. Symptoms associated with the presence of CAN were syncope (*p* = 0.000), postprandial nausea (*p* = 0.042), early satiety (*p* = 0.031), sexual dysfunction (*p* = 0.049), and gustatory sweating (*p* = 0.018).

**Table 4:** **Prevalence of autonomic symptoms in patients with and without CAN**.

Autonomic symptoms	Patients			*p*
	Total	Presence of CAN	Absence of CAN	
Syncope, *n* (%)	25 (16.6)	15(32.6)	10(9.5)	0.000
Postprandial nausea, *n* (%)	20 (13.2)	10 (21.7)	10 (9.5)	0.042
Early satiety, *n* (%)	30 (19.9)	14 (30.4)	16 (15.2)	0.031
Diarrhea, *n* (%)	5 (3.3)	4 (8.7)	1 (1)	0.051
Constipation, *n* (%)	43 (28.5)	17 (37)	26 (24.8)	0.126
Recurrent urinary infection, *n* (%)	9 (6)	5 (10.9)	4 (3.8)	0.189
Pollakiuria, *n* (%)	6 (4)	3 (6.5)	3 (2.9)	0.543
Sexual dysfunction, *n* (%)	34 (22.5)	15 (32.6)	19 (18.1)	0.049
Gustatory sweating, *n* (%)	16 (10.6)	9 (19.6)	7 (6.7)	0.018
Asymptomatic hypoglycemia, *n* (%)	55(44.7)	15 (39.5)	40 (47)	0.434

*CAN, cardiovascular autonomic neuropathy*.

The clinical and laboratory predictors of CAN found in the studied population were age (*p* = 0.01), diabetes duration (*p* = 0.036), hypertension (*p* = 0.048), resting HR (*p* = 0.000), HbA1c (*p* = 0.048), urea (*p* = 0.000), creatinine (*p* = 0.008), GFR (*p* = 0.000), urinary albumin concentration (*p* = 0.000), LDL-cholesterol (*p* = 0.048), free T4 (*p* = 0.023), and hemoglobin (*p* = 0.01) (Table 5). All microvascular chronic complications were related to the presence of CAN (Table 5). Only 1 (2.2%) patients with CAN presented levels of HbA1c lower than 7% compared with 18 (17.2%) in patients without CAN (*p* = 0.011).

**Table 5:** **Predictors of CAN in the studied population**.

	Presence of CAN	Absence of CAN	*p*
Female/male (%)	65.2/34.8	48.6/51.4	0.059
Caucasian/not Caucasian (%)	39.1/60.9	56.2/47.6	0.284
Age (years)	37.5 ± 12.7	31.6 ± 12.8	0.010
Age at diagnosis (years)	19.9 ± 9	16.4 ± 10.1	0.137
T1D duration (years)	18.5 ± 8.7	15.3 ± 9.8	0.051
T1D duration (<5/5–10/10–20/>20 years) (%)	4.4/15.2/39.1/41.3	11.4/23.8/44.7/37.6	0.036
Smokers, yes (%)	10.9	5.7	0.434
Alcohol consumption, yes (%)	19.6	16.2	0.631
BMI (Kg/m^2^)	22.7 (16.7–37.9)	23.6 (13.7–33.6)	0.505
Overweight/obesity, yes (%)	30.4	37.1	0.464
sBP (mmHg)	127.6 ± 19.7	123.3 ± 14.9	0.145
dBP (mmHg)	76.3 ± 10.4	74.5 ± 10.5	0.345
Hypertension, yes (%)	41.3	16.2	0.001
Resting heart rate (bpm)	87.3 ± 15.6	76.1 ± 11.1	0.000
Abdominal circumference (cm)	78 ± 9.6	80.6 ± 9.8	0.140
Fasting glycemia (mg/dl)	154 (44–364)	132 (24–548)	0.418
Postprandial glycemia (mg/dl)	240.2 ± 103.6	204.8 ± 96.5	0.056
HbA1c (%)	9.8 ± 2.4	8.9 ± 2.2	0.048
HbA1c <7%, *n* (%)	1 (2.2)	18 (17.1)	0.011
Urea (mg/dl)	34 (4–85)	27 (14–60)	0.000
Creatinine (mg/dl)	1.1 (0.52–2.68)	0.96 (0.44–1.45)	0.008
Uric acid (mg/dl)	4.8 ± 1.3	4.3 ± 1.2	0.055
Total cholesterol (mg/dl)	190 (117–330)	171 (110–193)	0.051
Triglycerides (mg/dl)	79 (37–320)	79 (32–507)	0.539
HDL-c (mg/dl)	65 (39.4–165.8)	62.9 (32.7–112.2)	0.563
LDL-c (mg/dl)	99.1 ± 25.2	89.7 ± 26.6	0.048
Dyslipidemia, yes (%)	45.7	29.5	0.055
CRP (mg/dl)	0.2 (0.01–4.33)	0.13 (0.01–3.4)	0.523
CRP (<1/1–3/>3 mg/dl), (%)	77.3/18.2/4.5	85.1/8.9/5.9	0.275
TSH (μUI/ml)	2.34 (1.35–6.93)	1.66 (0.32–5.61)	0.134
Free T4 (μUI/ml)	1.3 ± 0.3	1.2 ± 0.2	0.023
Hemoglobin (g/dl)	12.7 ± 1.6	13.4 ± 1.5	0.010
Urinary albumin concentration (mg/dl)	15.5 (1.77–152.3)	8.1 (0.1–257.3)	0.000
GFR (ml/min/1.73 m^2^)	86.8 ± 38.6	109.9 ± 35.8	0.000
Abnormal GFR (<60 ml/min/1.73 m^2^) (%)	19.6	5.7	0.009
Retinopathy, yes (%)	76.7	29.4	0.000
Nephropathy, yes (%)	47.7	13.9	0.000
Neuropathy, yes (%)	58.7	16.2	0.000
Coronary artery disease, yes (%)	6.5	3.8	0.757
Hypothyroidism, yes (%)	8.7	9.5	0.872

*T1D, type 1 diabetes mellitus; BMI, body mass index; sBP, systolic blood pressure; dBP, diastolic blood pressure; HbA1c, glycated hemoglobin; HDL-c, high density lipoprotein cholesterol; LDL-c, low-density lipoprotein cholesterol; CRP, C-reactive protein; TSH, thyroid stimulating hormone*.

*Values are presented as mean ± SD and median (minimum–maximum)*.

After logistic regression model using diagnosis of CAN by cardiovascular reflex testes as a dependent variable and sex, age, diabetes duration, pulse pressure (calculated as the difference between the sBP and dBP), resting HR, BMI, hypertension, albuminuria, presence of diabetic neuropathy and retinopathy, HbA1c, urea, creatinine, LDL, and hemoglobin as independent variables, it was observed that only resting HR, presence of diabetic neuropathy and retinopathy were significantly independent variables associated with the diagnosis of CAN (Table 6).

**Table 6:** **Logistic regression with the diagnosis of cardiovascular reflex tests as the dependent variable**.

	*B*	p*	OR	IC (95%)
Resting heart rate	0.124	0.001	1.13	1.06–1.22
Diabetic neuropathy	1.913	0.011	6.77	1.56–29.4
Diabetic retinopathy	1.939	0.008	6.95	1.68–28.8

*p < 0.05; OR, odds ratio; CI, confidence interval of 95%; dBP, diastolic blood pressure.

## Discussion

In the present study, the prevalence of incipient, defined, and total CAN in patients with T1D and T1D duration ≥5 years was 19.2, 11.3, and 30.5%, respectively when used only cardiovascular reflex tests; 6.6, 33.8, and 40.4% when using the HRV parameters (spectral analysis) and 9.3, 25.8, and 35.1% when used both criteria. The prevalence of CAN assessed by cardiovascular reflex tests was lower than the prevalence reported in the literature (11.3 versus 25%), but quite similar when using cardiovascular reflex tests associated with the HRV parameters (spectral analysis) (25.8 versus 25%). As we can see, the prevalence of CAN defined by HRV (spectral analysis) was superior to cardiovascular reflex tests, which could corroborate the data from the literature regarding the sensitivity and early detection of cardiovascular autonomic dysfunction by this diagnostic criterion. However, the diagnosis of CAN based only on spectral analysis must be carefully considered since previous study from our group (19) demonstrated that the assessment of HRV by spectral analysis (VLF, LF and HF) showed low reproducibility.

The agreement of CAN diagnosis for the two diagnostic methods (cardiovascular reflex tests versus spectral analysis) was moderate. Therefore, analysis of CAN using only frequency-domain HRV analysis should be considered an additional tool for CAN diagnosis according to other studies (16–18, 36).

The incidence of CAN increases with age, diabetes duration and poor glycemic control. (7,8) In this study, clinical and laboratory predictors of CAN were age, T1D duration >10 and 20 years, hypertension, resting HR, HbA1c, LDL-c, urea, creatinine, free T4 and hemoglobin, and the presence of retinopathy, nephropathy and diabetic peripheral neuropathy. Slightly higher levels of free T4, even within the normal range were found in patients with T1D and CAN, possibly suggesting a sympathovagal imbalance (16).

Some studies showed that CAN can reduce the renal release of erythropoietin probably by renal denervation, leading to the development of anemia (37, 38). In our study, we observed that patients with T1D and CAN had lower hemoglobin levels, corroborating the above mentioned data. Patients with T1D and proteinuria may have anemia by erythropoietin deficiency, even in the absence of changes in GFR (38).

However, it is difficult to differentiate whether the anemia was a result of CAN or diabetic nephropathy since these two diabetic complications are frequently associated.

Several studies have suggested a causal relationship between CAN and diabetic nephropathy (39, 40). Forsen et al (39) evaluated the prevalence of microalbuminuria and CAN after 14 years of follow-up and observed that CAN precedes the development of albuminuria in patients with T1D. In patients with T1D and CAN, the sympathovagal imbalance (assessed by abnormal E:I ratio) promotes increase in nocturnal BP (reduction of parasympathetic activity) with consequent overnight increase in intraglomerular pressure, and daytime reduction in intraglomerular pressure as a result of postural hypotension (39, 41). CAN appears to be a permissive factor for the development of persistent microalbuminuria (39). In our study urinary albumin concentration and GFR were associated with the presence of CAN, corroborating the association of these two complications. This study was a cross-sectional study and could not establish a causal relationship between diabetic nephropathy and CAN.

Inflammatory markers such as CRP and IL-6 have been correlated with abnormal HRV (6). One study evaluated 611 healthy subjects and showed an inverse correlation between CRP levels and HRV parameters (42). Ridker et al (43, 44) have shown an association of CRP levels >3 mg/dl with high cardiovascular risk. In the current study, we found no statistically significant association between CRP levels and CAN, which could be explained by the fact that only 8 patients had CRP levels ≥3 mg/dl.

Autonomic symptoms, although disabling, appears in more advanced stages of CAN, have low sensitivity and specificity and have low association with abnormal cardiovascular reflex tests (6, 17, 18). Symptoms associated with CAN in this study symptoms were syncope, postprandial nausea, early satiety, sexual dysfunction and gustatory sweating. Therefore, autonomic symptoms did not seems to represent sensitive indicators for early diagnosis of CAN (16).

This study is one of the first studies that evaluated the prevalence of CAN assessed by the gold standard cardiovascular reflex tests as recommended by the ADA in the Brazilian population, thus allowing the identification of patients with T1D and increased cardiovascular risk. Moreover, this study allowed the identification of some clinical factors that might suggest the diagnosis of CAN.

The study had some limitations. Because this was a cross-sectional study we could not establish a causal relationship between the predictors and the diagnosis of CAN. Another limitation is related to data collection on hypoglycemia. Patients self-reported the number and severity of hypoglycemic episodes in the last month. It was not possible to obtain data through the glucometer of all patients, which could underestimate the prevalence of hypoglycemia.

In conclusion, CAN is a common chronic complication of T1D affecting about 30% of the studied population and is associated with the presence of other chronic complications of T1D. Indicators of the presence of CAN included age, duration of diabetes, presence of hypertension, resting HR, presence of diabetic neuropathy and retinopathy and symptoms suggestive of autonomic neuropathy. This study confirms the importance of systematic and early screening for this complication.

## Author Contributions

Lucianne Righeti Monteiro Tannus and Marilia Brito Gomes researched data, drafted the manuscript. Karla Rezende Guerra Drummond, Eliete Leão da Silva Clemente, and Maria de Fátima Bevilacqua da Matta contributed to the conception of the work and the acquisition of data for the work. Lucianne Righeti Monteiro Tannus and Marilia Brito Gomes reviewed the manuscript and contributed to the discussion. The writing group takes final responsibility for the paper and is the study guarantor.

## Conflict of Interest Statement

The authors declare that the research was conducted in the absence of any commercial or financial relationships that could be construed as a potential conflict of interest.
